# COVID-19 response in northwest Syria: innovation and community engagement in a complex conflict

**DOI:** 10.1093/pubmed/fdaa068

**Published:** 2020-05-21

**Authors:** Abdulkarim Ekzayez, Munzer al-Khalil, Mohamad Jasiem, Raed Al Saleh, Zedoun Alzoubi, Kristen Meagher, Preeti Patel

**Affiliations:** 1 Conflict and Health Research Group and R4HC-MENA, King’s College London, UK; 2 Syrian Public Health Network, London, UK; 3 Idleb Health Directorate, Idleb, Syria; 4 Early Warning and Response Network (EWARN), The Assistance Coordination Unit, Gaziantep, Turkey; 5 White Helmets (Syria Civil Defence), Idleb, Syria

**Keywords:** infectious disease, population-based and preventative services, war, COVID-19, Syria, Idleb, Surveillance, Social distancing

## Abstract

Despite lacking capacity and resources, the health system in the northwest Syria is using innovative approaches for the containment of COVID-19. Lessons drawn from previous outbreaks in the region, such as the polio outbreak in 2013 and the annual seasonal influenza, have enabled the Early Warning and Response Network, a surveillance system to develop mechanisms of predicting risk and strengthening surveillance for the new pandemic. Social media tools such as WhatsApp are effectively collecting health information and communicating health messaging about COVID-19. Community engagement has also been scaled up, mobilizing local resources and encouraging thousands of volunteers to join the ‘Volunteers against Corona’ campaign. Bottom-up local governance technical entities, such as Idleb Health Directorate and the White Helmets, have played key leadership role in the response. These efforts need to be scaled up to prevent the transmission of COVID-19 in a region chronically affected by a complex armed conflict.

## Introduction

As of 1 May 2020, the COVID-19 pandemic has hit 214 countries around the world with 3 175 207 confirmed cases and 224 172 deaths.^1^ While countries with the most advanced health systems are struggling to fight COVID-19, conflict-affected countries are facing potentially devastating outbreaks.^2^ The International Crisis Group recently stated that it was ‘especially concerned with places where the global health challenge intersects with wars or political conditions that could give rise to new crises or exacerbate existing ones’.^3^ Extremely weak health systems, mass displacement and lack of basic infrastructure are all factors that place areas affected by armed conflict, such as Yemen, Afghanistan and several countries in sub-Saharan Africa, at a much higher risk for COVID-19 pandemic.^4^^,^^5^

The health system in the northwest Syria has faced enormous challenges throughout the 9 years of the Syrian conflict. Daily attacks on health workers and facilities, collapse of central governance, lack of resources and supplies and a rapidly changing humanitarian, political and military environment have contributed to a weak health system in a region of around 3.5 million population.^6–9^ Currently, the health system of the northwest Syria has around 1.4 doctors per 10 000 people, ~0.625 hospital beds per 1000 people, ~5.7 intensive care unit (ICU) beds per 100 000 people—but with only 47 functioning adults-ventilators for the whole region. As a result, the health system is neither prepared nor capable to deal with the current increasing health needs, let alone the predicted spread of the COVID-19.

This health system has survived nearly a decade of civil war but now faces a new kind of enemy as the impact of a possible spread of COVID-19 in the northwest Syria will be catastrophic.^10^ Factors that put the estimated 3.5 million population at a higher risk include (i) the presence of >2.8 million internally displaced persons; (ii) overcrowding in all urban and rural areas as well as in the 500+ arbitrary camps in the region;(iii) the extreme poverty rate with ~83% of Syrians living below the poverty line; (iv) the intense social mixing and (v) the large average household size.^4^^,^^11–13^

The resilience of local communities and the health system in the northwest Syria has weakened considerably during the last few months with the relentless military offensive by the Government of Syria and its allies, which began in December 2019. This has forcibly displaced around 1 million people towards the closed Turkish border in the north,^13^ with >68 health facilities attacked between April 2019 and February 2020.^14^ The extension of UN Security Council Resolution 2165 (2014) for cross-border aid was vetoed by Russia and China, which has considerably reduced access for humanitarian aid and other essential supplies.^15^ And worryingly, the crossing points at the Syrian–Turkish border were reduced to only two points and for a period of only 6 months—which seriously hampers access to the northwest Syria.^16^

As of 28 April 2020, there have been 43 confirmed cases of COVID-19 in the Government held areas,^17^ and no confirmed cases of COVID-19 reported in the northwest Syria.^18^ However, the local health authorities in the northwest Syria transparently say that they cannot confirm that the region is free of cases given the limited testing capacity. Northwest Syria is naturally isolated from surrounding territories because of the geopolitical situation, which means population movements into this region are very limited compared to the other areas of Syria. This natural isolation gives the region some protective advantage that might delay the onset of the outbreak, and many experts believe this time should be invested in scaling up the preparedness and emergency planning.

## Key features of the COVID-19 response in the northwest Syria

With increased global attention on the importance of preparedness and emergency planning for the COVID-19 response, the health system in the northwest Syria has taken practical steps to prepare for the response against a backdrop of very limited resources.

### Governance and leadership

Northwest Syria is an opposition-held region where several local groups and de facto authorities dispute power and control. At least four main rival military factions with many other smaller armed groups control this region.^19^ In such a complex situation, it is almost impossible to establish a well-coordinated central response. Since 3 March 2020, the World Health Organization (WHO)-led health cluster has established a COVID-19 task force that consists of local and international NGOs. The task force has developed basic emergency planning for potential scenarios alongside technical guidelines.^20^ However, WHO has limited capacity to engage in such a large-scale complex conflict environment as it does not have any physical presence inside Syria and has been facing enormous geopolitical challenges for its cross-border response from Turkey. The Idleb Health Directorate (IHD) throughout its 7 years of existence has maintained its position as the technical health authority in the region. To navigate through this environment, IHD has created a grassroots governance system that enabled its legitimacy to be derived from all medical doctors in the area.^21^ Legitimacy and trust are important factors in getting population to follow IHD proposed guidelines in relation to COVID-19 response. Gaining trust and legitimacy are important lessons from the previous polio outbreak in Syria and from the Ebola outbreak in West Africa.^22^^,^^23^

### Community engagement and mobilizing local resources

However, the COVID-19 response necessitates more executive power with decentralized mobilization of resources. Thus, IHD, alongside with many local actors including the White Helmets or Syria Civil Defence, called for a mass voluntary campaign inviting local groups to take part in the response. This campaign, ‘Volunteers against Corona’ has mobilized thousands of volunteers covering most localities in the region.^24^^,^^25^ The campaign organizes volunteers in various technical teams and neighbourhood committees. The technical teams cover tasks related to raising awareness, disinfection campaigns and community-based referrals. Neighbourhood committees are responsible for raising awareness in their localities, identifying high risk groups to shield them and linking local communities with the central campaign. The campaign utilizes social media, free cloud web servers and WhatsApp communication to communicate efficiently with this large group of volunteers. Facebook is used as the hub for all volunteer groups to obtain updates on the campaign and on technical guidelines, and WhatsApp is used for all day-to-day communication and for team management purposes.

### The role of diaspora network in managing knowledge

As SARS-CoV-2 virus is a new pathogen, all related knowledge generation is novel—this means time is required to acquire the latest evidence, verify its effectiveness and disseminate this through public health policies. The response so far has involved a wide network of Syrian medical diaspora especially in the UK and France. These networks have been utilized to provide the local health system with the latest evidence on the virus. A central medical chat room has been established on WhatsApp where relevant information is shared on daily basis. Several online remote training sessions are provided using a variety of online platforms including Skype, Zoom, GoToMeeting and Google Meet. In addition, a repository of resources, educational materials and training packages have been established—these are used by field health workers in remote areas of the region.

### Learning from the polio outbreak in 2013

The polio outbreak in October 2013 was the first major public health threat that tested the readiness and responsiveness of the health system in the northwest Syria. The polio response necessitated establishing core vaccination structures with wide networks of volunteers to reach every household in the region. The Polio Control Task Force was the first of its kind, which resulted from enormous coordination efforts between UN agencies, the WHO and international and local NGOs. The task force led the response effectively with support from different donors. In addition, considerable investment was made for local vaccination structures, linking them with the Health Directorates as central coordination bodies.^23^^,^^26^^,^^27^ A similar approach has been adopted for the COVID-19 response with a task force operating from Turkey, a central field coordination mechanism in Idlib, and investment in local volunteers.

### SARS-CoV-2 surveillance: a proactive risk assessment

Diseases surveillance in all territories outside the control of the Syrian government is covered by the Early Warning and Response Network (EWARN) that was established by a Syrian NGO ‘the Assistance Coordination Unit’ in 2013 with limited resources.^28^ Shortly after its establishment, the EWARN was able to share the initial alert for the first case of polio in eastern Syria in October 2013.

A year ago, there was no polymerase chain reaction (PCR) testing capability inside the northwest Syria. However, since 2017, EWARN has scaled up capacity to identify pathogens of two tracked diseases: severe acute respiratory infection and influenza-like illness. With technical and financial support from the Gates Foundation, EWARN has requested WHO to supply required training and equipment to prepare a reference lab for PCR testing inside Syria. As a result, with the start of this flu season (September 2019), the EWARN has started preparing for a flu-like outbreak. This preparation has meant that the EWARN has basic capacity to deal with the first phase of COVID-19 response. As early as November and December 2019, the EWARN has identified an outbreak of H1N1 in northern Aleppo. In response, the EWARN has started to scale up testing capabilities for flu-like illness.

With the start of the COVID-19 pandemic, the EWARN ran out of the only 300 PCR tests that they had previously running 191 tests. They relied on a Turkish reference lab to supply required materials and trainings to conduct a very limited number of tests. By mid-April, EWARN have received another shipment from WHO with 5000 tests. As of 22 April 2020, 191 tests of COVID-19 have been run across the northwest Syria.^18^ Currently, the EWARN is gradually scaling up SARS-CoV-2 testing inside Idleb region to reach a target of 100 tests a day and there are plans to establish two additional reference labs inside the northwest Syria to scale up with the testing capacity.

The EWARN is not only responsible for testing, it covers a wide range of epidemiological roles that include contact tracing and risk mapping and other public health measures related to hygiene, health promotion, water and sanitation. Although WHO has had no clear plans for strengthening local surveillance capacity in northern Syria, the EWARN has been proactive in predicting risks and potential outbreaks.

### Key preventative and community engagement measures

In light of the limited capacity of the health system in the northwest Syria to deal with a possible expansion of cases, the focus has been on preventative measures that contain and delay the spread of the virus. These measures are summarized in Table 1.

**Table 1:** Key COVID-19 preventative measures in the northwest Syria

Measure	What have been done so far
Control of borders and crossing points	All official crossing points with the Government of Syria and the AANES areas have been closed from mid-March 2020. The Bab Al Hawa border with Turkey has been restricted from the Turkish side with very minimum cross-border activities for trade and humanitarian supplies.
Social distancing	All health actors have been asking people to stay at home, where possible, and reduce social gatherings and events. All schools were closed. However, this measure has been challenging considering the high poverty rate, high population and household density, some social and cultural practices that involve high number of people such as funerals and congregational prayer. Engaging with various local actors including the local councils, community and religious leaders was key to overcome some of these challenges.
Public awareness campaigns	Health and local NGOs have engaged in various public awareness activities including distribution of more than a million educational materials—such as leaflets and brochures, household visits in camps and radio messaging.
Disinfection campaigns	These campaigns targeted mainly the residential collective centres, camps, public buildings such as schools and health facilities. The White Helmets played a key role in conducting these campaigns with disinfecting >5000 sites on regular basis.
Quarantine and isolation	IHD has started a project to establish 17 community-based isolation centres with a capacity of 1400 beds that are expected to be ready in the first week of May.

### Scaling up health system capacity using limited resources

Working with the health cluster task force and with diaspora medical networks in France and the UK, IHD has developed a clinical response plan to scale up the health services using limited resources. The plan prioritizes scaling up ICU capacity to set up more ventilators where possible with the aim of responding to the predicted 14% of possible patients who would develop severe respiratory complications. Clear clinical definitions of suspected and confirmed cases were developed alongside with clinical standards of procedures on how to triage, refer, workup diagnosis and possible treatment protocols. The task force recommended establishing three new hospitals for COVID-19 patients. However, no funding was available to implement this plan. IHD has prepared one department in the main district hospital in Idleb district to receive all suspected cases. Further resources are needed to scale up inpatients and ICU capacity.

Infection prevention and control (IPC) was at the core of the response planning. Triage tents in front of most health facilities were set up to ensure suspected patients do not infect other patients and routine health services are not interrupted. The IPC guidelines and protocols have been reviewed and updated to reflect latest evidence. In addition, >500 medical personnel were trained on IPC. However, the lack of resources is hindering the health system scale-up plans.

### Resource-limited digital solutions

The first author of this paper was involved in developing an Arabic speaking website for self-assessment for COVID-19 targeting the population of the northwest Syria.^29^ The website collects information on (i) users’ location—on subdistrict level, (ii) travel history, (iii) suspected signs and symptoms, (iv) risk factors and (v) the presence of severe complications. Based on this information, an algorithm classifies users into five categories depending on the level of disease suspicion and the presence of any risk factor or server complication that might require immediate attention. Accordingly, users will be advised to follow certain recommendations or access certain services.

The website was developed in coordination with a local Syrian NGO called ‘Violet Syria’, which has been involved in various social mobilization activities related to COVID-19. The organization has coordinated with local groups to advise communities to use the website for initial self-assessment. The aim was to reduce pressure and overload on the already-stretched health system in the region.

We plan to develop this initiative further and collect more information systematically on suspected symptoms and risk factors with the ability to map out these data. This mapping will help in understanding the prevalence of suspected cases. Such risks mapping will help in allocating the scarce health resources in these settings.

Social media and communication tools have been recruited effectively in collecting health information and communicating health messaging. IHD has dedicated a WhatsApp number to respond to queries from public on COVID-19. Diaspora networks have also been involved in providing remote consultations and support to members of the public as well as to their medical colleagues on the ground. Facebook has been used to organize the volunteer campaigns and to convey messages considering the wide use of Facebook in the northwest Syria.

**Key points**

- Despite lacking capacity and resources, the health system in the northwest Syria is using innovative approaches for the containment of COVID-19. These efforts should be supported to scale up the capacity of the health system shall the virus spread in this region.
- Learning from previous outbreaks, such as polio 2013 and seasonal influenza, was an important factor in predicting risk and strengthening surveillance.
- Social media tools such as WhatsApp and newly developed websites are being used effectively to collect health information and communicating health messaging.
- The focus on community engagement have mobilized local resources and encouraged thousands of volunteers to join the ‘Volunteers against Corona’ campaign.
- Bottom-up local governance technical entities, such as Idleb Health Directorate and the White Helmets, played key leadership role in the response.
- The current health system indicators in the northwest Syria are alarming with only ~1.4 medical doctor per 10 000 people, ~0.625 hospital beds per 1000 people, <5.7 intensive care unit beds per 100 000 people and only 47 functioning adult ventilators for the whole region.
- Protection of health workers should be a core principle in designing the COVID response in the northwest Syria. Any loss for any medical human resources is irreversible.
- The required resources include personal protective equipment, funding for new health infrastructure, oxygen supplies, ventilators and running cost for the health facilities.
- World Health Organization should mobilize more resources to scale up the capacity of the health system in the northwest Syria.

## Authors’ contributions

The theoretical framing, initial analysis and drafting and multiple rounds of edits were carried out by A.E. M.K., M.J., Z.A., R.S. and K.M. all contributed to the initial analysis with substantial information. P.P. has contributed to the theoretical framing and multiple rounds of editing.

## Funding

This research was funded through UK Research and Innovation as part of the Global Challenges Research Fund; Research for Health in Conflict in the Middle East and North Africa (R4HC-MENA) project (grant number ES/P010962/1).

## Ethics approval and consent to participate

Not applicable.

## Competing interests

The authors declare that they have no competing interests.

## Notes

**Abdulkarim Ekzayez,** Research Associate

**Munzer al-Khalil,** Health Director

**Mohamad Jasiem,** EWARN Manager

**Raed Al Saleh,** Director

**Zedoun Alzoubi,** Research Consultant

**Kristen Meagher,** Research Associate

**Preeti Patel,** Reader in Global Health and Conflict
